# The Combined Anti-Tumor Efficacy of Bioactive Hydroxyapatite Nanoparticles Loaded with Altretamine

**DOI:** 10.3390/pharmaceutics15010302

**Published:** 2023-01-16

**Authors:** Yahia Alghazwani, Krishnaraju Venkatesan, Kousalya Prabahar, Mohamed El-Sherbiny, Nehal Elsherbiny, Mona Qushawy

**Affiliations:** 1 Department of Pharmacology, College of Pharmacy, King Khalid University, Abha 62529, Saudi Arabia; 2 Department of Pharmacy Practice, Faculty of Pharmacy, University of Tabuk, Tabuk 71491, Saudi Arabia; 3 Department of Basic Medical Sciences, College of Medicine, AlMaarefa University, Riyadh 13713, Saudi Arabia; 4 Department of Anatomy and Embryology, Faculty of Medicine, Mansoura University, Mansoura 35516, Egypt; 5 Department of Pharmaceutical Chemistry, Faculty of Pharmacy, University of Tabuk, Tabuk 71491, Saudi Arabia; 6 Department of Biochemistry, Faculty of Pharmacy, Mansoura University, Mansoura 35516, Egypt; 7 Department of Pharmaceutics, Faculty of Pharmacy, University of Tabuk, Tabuk 71491, Saudi Arabia; 8 Department of Pharmaceutics, Faculty of Pharmacy, Sinai University, Alarish 45511, Egypt

**Keywords:** altretamine, cancer therapy, sustained delivery, hydroxyapatite nanoparticles, chemical precipitation method

## Abstract

In the current study, the combined anti-tumor efficacy of bioactive hydroxyapatite nano- particles (HA-NPs) loaded with altretamine (ALT) was evaluated. The well-known fact that HA has great biological compatibility was confirmed through the findings of the hemolytic experiments and a maximum IC_50_ value seen in the MTT testing. The preparation of HA-NPs was performed using the chemical precipitation process. An in vitro release investigation was conducted, and the results demonstrated the sustained drug release of the altretamine-loaded hydroxyapatite nanoparticles (ALT-HA-NPs). Studies using the JURKAT E6.1 cell lines MTT assay, and cell uptake, as well as in vivo pharmacokinetic tests using Wistar rats demonstrated that the ALT-HA-NPs were easily absorbed by the cells. A putative synergism between the action of the Ca^2+^ ions and the anticancer drug obtained from the carrier was indicated by the fact that the ALT-HA-NPs displayed cytotoxicity comparable to the free ALT at 1/10th of the ALT concentration. It has been suggested that a rise in intracellular Ca^2+^ ions causes cells to undergo apoptosis. Ehrlich’s ascites model in Balb/c mice showed comparable synergistic efficacy in a tumor regression trial. While the ALT-HA-NPs were able to shrink the tumor size by six times, the free ALT was only able to reduce the tumor volume by half.

## 1. Introduction

Apoptosis is a fundamental biological process that multicellular organisms use to replace aging, damaged, or unhealthy cells. Caspases (protein-digesting enzymes) are activated by an extrinsic or intrinsic mechanism, initiating a tightly controlled process that ultimately results in cell death. TNF-α, and Fas are examples of extracellular ligands that begin the extrinsic pathway, whereas stress-induced intracellular signals start the intrinsic pathway [1]. One of the mechanisms that stimulate intracellular apoptotic signaling is increased intracellular calcium ion (Ca^2+^) concentrations. Cancerous cells are currently being treated with chemotherapy employing substances that interfere with the calcium ion homeostasis in cells. Kim et al. examined the effects of panaxydol, which was extracted from Panax Ginseng roots, on MCF-7 (human breast cancer) cells. They discovered that the substance might cause cell death and raise intracellular Ca^2+^ ion concentrations [2]. Nagoor Meeran et al. investigated the effects of carvacrol, a phenol-derived (monoterpenoid) from origanum vulgare, on human glioblastoma cells. Carvacrol had a raised intracellular Ca^2+^ ion concentration, promotes Ca^2+^ release from the ER store, is phospholipase C-dependent, and results in cell death [3]. Apoptosis has also been shown to be induced via a similar method by other substances of natural sources, such as Cryptotanshinone (a tanshinone isolated from the root of *Salvia miltiorrhiza*) and Diospyrin (a naphthoquinone isolated from the stem bark of Diospyrosmontana). Apoptosis is known to be induced similarly by synthetic substances including Auranofin, Vismodegib, and GaQ(3) (KP46), a new gallium complex. The testing of these substances is still in its early stages. The significant expenses involved with their production/isolation further restrict their medicinal value. Utilizing the NPs of calcium phosphates and hydroxyapatite (HA) is one of the practical options for increasing the Ca^2+^ content of the interior part of the cell [4]. HA is a substance that is abundant in nature and is biologically active, particularly in the calcified tissue of vertebrates. It has been utilized as a transporter when creating delivery systems for medicinal agents such as medicines, enzymes, antigens, DNA, and other proteins. Additionally, it has been discovered that hydroxyapatite nanoparticles have a proapoptotic and antiproliferative effects on malignant cells [5,6]. In their study of the anticancer inhibitory impact of HA, Zhang et al. found that the treatment with HA significantly inhibited cancer cells when compared to normal cells [7]. Similar research was conducted by Tang et al., who concluded that the increased intracellular calcium ion concentration may be the cause of the inhibitory effect of HA [8].

This investigation proposes the usage of altretamine (ALT)-loaded HA-NPs for synergistic anticancer action based on the above explanation. Once in the cell, the HA-NPs will progressively break down, releasing the medication over time while also raising the Ca^2+^ concentration there. The methyl melamine class of these compounds includes the synthetic alkylating agent known as altretamine (ALT). The alkylating chemicals work by altering and cross-linking DNA, which prevents the production of DNA, RNA, proteins, and results in the cell death of rapidly proliferating cells [9]. To evaluate the synergistic anticancer potential of this combination, acute T cell leukemia cells, JURKAT E6-1 cells, in vivo pharmacokinetic experiments in Wistar rats, and tumor regression studies in Balb/c mice utilizing the Ehrlich’s ascites model were also employed.

Calcium levels are controlled at 400–600 mM in lysosomes and 200–400 nM in the cytosol. In cellular metabolism, even small variations in intracellular calcium concentration have a significant impact. The pH of lysosomes and endosomes will rise as the amounts of calcium and phosphate ions increase [10]. The compartment will become Ca^2+^ supersaturated, which will prevent HA-NPs from dissolving further. This may be the reason why altretamine-loaded hydroxyapatite nanoparticles (ALT-HA-NPs) are additionally effective after 72 h of treatment, owing to the comparatively slow dissolution of HA-NPs, which results in an improved penetration and retention effect. High endosomal or lysosomal calcium ions allow HA-NPs to bypass the phagocytic route and move directly into the cell nucleus where they can infect it. An increase in calcium ions up-regulates the movement of particles through nuclear pores and their escape from phagocytic pathways. The rate of programmed cell death is accelerated by the intracellular Ca^2+^ concentration [11]. DNA had expurgated by calcium-dependent endonucleases when Ca^2+^ is present. Intracellular calcium ions promote the permeabilization of the mitochondrial sheath and the release of proapoptotic mitochondrial protein, i.e., cytochrome C. The endoplasmic reticulum releases more calcium ions, which in turn releases more cytochrome C from the mitochondria. This is the result of the original release of cytochrome C. The Apoptosome was formed by further combining Cytochrome C with Pro-Caspase-9, ATP, and Apoptotic Protease Activating Factor-1. The effector Caspase-3 is activated when the Pro-Caspase is broken down by the Apoptosome into its active form, Caspase-9. The apoptosis process was then carried out by the active effector caspases digesting a variety of intracellular proteins. Additionally, microtubules typically work more efficiently in ATP-dependent ways in situations of high intracellular calcium, which results in ATP depletion and is a well-known sign of apoptosis. Additionally, the cytoskeleton is disrupted in the vacuoles that hold HA-NPs in the cytoplasm. In other words, it is believed that the presence of calcium content activates the Calpain, as Ca^2+^-dependent protein kinases disrupt the cytoskeleton. This results in the disruption of the cytoskeleton around HA-NPs. Additionally, the high surface energy of HA-NPs due to the abundant Ca^2+^ ions can stimulate certain molecules and create free radicals. Actin filament damage caused by ROS can compromise the integrity of the cytoskeleton and cause cytoskeleton disruption [12]. Ca^2+^ can also activate NO synthases, transglutaminase, calcineurin, endonucleases, or phospholipases. These substances have been demonstrated to play a role in the different types of apoptotic cell death. The role of calcium-binding proteins including ALG-2 or calcium/calmodulin-dependent kinases such as the death-associated protein kinase and other enzymes known to be vital in apoptosis are only a few examples of other pathways that have been reported [13].

## 2. Materials and Methods

### 2.1. Materials

Altretamine (ALT) was received from Spectra Lab. Hyderabad, India, as a gift sample. HiMedia Lab Pvt. Ltd., Mumbai, India provided the calcium nitrate tetrahydrate (Ca(NO_3_)_2_.4H_2_O), ammonium dihydrogen phosphate (NH_4_H_2_PO_4_), ammonia solution, methanol, and isopropyl alcohol that was needed. HPLC-grade water, methanol, and acetonitrile were purchased from Sigma-Aldrich, Bangalore, India. Internally prepared Milli-Q water was used during the trials. There were also analytical-grade reagents employed.

### 2.2. Synthesis ALT-Loaded HA-NPs (ALT-HA-NPs)

HA-NPs were prepared by using the wet chemical precipitation approach [14]. Firstly, separate aqueous solutions of 0.025 M (Ca(NO_3_)_2_ and 0.025 M of (NH_4_)_3_PO_4_ were made using Milli-Q water. After that, the previously prepared Ca(NO_3_)_2_ solution in 500 mL of the three-necked round-bottom flask with a rosary-type condenser in the center neck was placed in a silicon oil thermal bath and kept at 90 °C under magnetic stirring conditions [15]. An aqueous (NH_4_)_3_PO_4_ solution and NH_4_OH solution were added dropwise via the left neck of the flask, and the pH was determined using a potentiometer mounted on the right neck of the flask. To produce HA particles, the solution was allowed to stir for 3 h until the required pH level was obtained [16]. Equation (1) depicts the chemical precipitation reaction for the production of HA. ALT was physically adsorbed onto the prepared HA-NPs.

6(NH_4_)_2_HPO_4_ + 10Ca(NO_3_)_2_. 4H_2_O + 8NH_4_OH → 20NH_4_NO_3_ +Ca_10_(PO_4_)_6_(OH)_2_ + 46H_2_O (1)

### 2.3. Determination of the Encapsulation Efficiency % and Loading Capacity % of ALT-HA-NPs

The encapsulation efficiency % (EE %) of ALT in the prepared HA-NPs was determined by an indirect method where 2 mL of the prepared nanoparticle dispersion was centrifuged at 15,000 pm for 45 min. The supernatant was separated and analyzed spectrophotometrically at 226 nm after appropriate dilution to determine the free ALT [17]. The encapsulation efficiency was calculated using Equation (2):

(2) $$\left(\mathrm{EE}\%\right)=\frac{\mathrm{Total}\;\mathrm{amount}\;\mathrm{of}\;\mathrm{ALT}-\mathrm{free}\;\mathrm{ALT}}{\mathrm{Total}\;\mathrm{amount}\;\mathrm{of}\;\mathrm{ALT}}\times 100$$

Additionally, the drug loading capacity (DL%) was calculated [18] using Equation (3):

(3) $$\left(\mathrm{DL}\%\right)=\frac{\mathrm{The}\;\mathrm{amount}\;\mathrm{of}\;\mathrm{loaded}\;\mathrm{ALT}\;\mathrm{in}\;\mathrm{nanoparticles}\;\left(\mathrm{mg}\right)}{\mathrm{weight}\;\mathrm{of}\;\mathrm{nanoparticles}\;\left(\mathrm{mg}\right)}\times 100$$

### 2.4. Particle Size Analysis of ALT-HA-NPs

The particle size of the prepared ALT-HA-NPs was determined by dynamic light scattering technique using zetasizer (Malvern Instruments Ltd., Malvern, UK). The sample was diluted to the appropriate concentration, and the measurements were carried out at a temperature of 25 °C and an angle of 90° [19].

### 2.5. Determination of Surface Morphology of ALT-HA-NPs

#### 2.5.1. Transmission Electron Microscopy (TEM)

The surface morphology was determined by TEM (JTEM model 1010, JEOL^®^, Tokyo, Japan). One drop of the prepared ALT-HA-NPs was added to the collodion-coated copper grid after being diluted with distilled water. The dried sample was stained with uranyl acetate solution and examined with TEM [20].

#### 2.5.2. Scanning Electron Microscopy (SEM)

A small amount of the dry powder of the prepared ALT-HA-NPs was coated with approximately 15 nm gold (SPI-Module Sputter Coater). The coated sample was scanned by an analytical scanning electron microscope (JSEM-6360LA, JEOL^®^, Tokyo, Japan) under vacuum conditions at 15 kV acceleration voltage at room temperature [21].

### 2.6. In Vitro Release Studies

The drug release study was performed using the dialysis method. Where 2 mL (3 mg equivalent of ALT) of the prepared ALT-HA-NPs was transferred into a dialysis bag (cut-off 12kDa, Sigma). The dialysis bag was immersed in a beaker containing 100 mL of Phosphate Buffer Saline (PBS; pH 7.4) as a dissolution medium. The dissolution medium was stirred at 100 rpm and kept at 37 ± 1 °C. The samples were withdrawn at different time intervals up to 24 h and analyzed spectrophotometrically at 226 nm after appropriate dilution. The experiment was performed in comparison with the free ALT in triplicate and the mean ± SD was calculated [22,23].

### 2.7. Hemolytic Activity Determination

A previously reported procedure was used to evaluate the hemolytic activity of whole blood. Briefly, heparinized blood was obtained from dependable sources (Research Ethics Review Board (ERB) approval no. 2022/20137/MedAll/TRY; dated 11-02-2022). Before the experiment, human red blood cells were twice washed in PBS (pH 7.4). In 96-well microtiter plates, 100 µL of human RBC was plated with 0.4% (*v*/*v*) in PBS suspension and subsequently incubated for up to 24 h at 37 °C with an equal amount of ALT, ALT-HP-NPs, normal saline (0% hemolysis) aided as the negative control, and Milli-Q water (100% hemolysis) aided as the positive control. Equation (4) is the formula used to compute the % hemolysis in the experiment, which was done in triplicate [24]. In an ELISA reader, the lysed RBC absorbance was measured at 540 nm. PBS and 1% Triton-X 100 were used to determine 0% and 100% hemolysis.

(4) $$\mathrm{Hemolysis}\;\left(\%\right)=\frac{\mathrm{Abs}\;\left(\mathrm{sample}\right)-\;\mathrm{Abs}\;\left(\mathrm{negative}\;\mathrm{control}\right)}{\mathrm{Abs}\;\left(\mathrm{positive}\;\mathrm{control}\right)-\mathrm{Abs}\;\left(\mathrm{negative}\;\mathrm{control}\right)}\times 100$$

### 2.8. In Vitro Cell Line Studies

#### Cytotoxicity Assessment

A previously described procedure was slightly modified to investigate the ALT, HA-NP, and ALT-HA-NP cytotoxicity. The MTT test on JURKAT cells was used to measure the percentage of cytotoxicity (E6-1; acute T cell leukemia cells). Briefly, the cells were seeded in a 96-well microtiter plate at a mass of 5 × 10^3^ cells·well^−1^, supplemented with fetal calf serum (2.5%), and then incubated at 37 °C for 24 h with a N_2_ atmosphere of 90% with 5% of CO_2_, and 5% O_2_ for 24, 48, and 72 h, respectively. The cells were treated with two concentrations of the samples, i.e., 150 µg·mL^−1^, and 300 µg·mL^−1^. For this addition of 20 µL of MTT solution (5 mg·mL^−1^ in PBS at pH 7.4) to each well subsequently, the required amount of time had passed. Then, DMSO (100 µL) was used to dissolve the formazan crystals [25]. At 540 nm, the optical density (OD) was determined from an ELISA microplate reader after mixing with a mechanical plate mixer (Synergy HT, BioTek, Winooski, VT, USA). Three copies of each measurement were made. After 24 h, IC_50_ values were collected for each sample, and the following equation (Equation (5)) was used to calculate the percentage of cytotoxicity:

(5) $$\mathrm{Toxicity}\;\left(\%\right)=\frac{\mathrm{Abs}\;\left(\mathrm{control}\right)-\;\mathrm{Abs}\;\left(\mathrm{sample}\right)}{\mathrm{Abs}\;\left(\mathrm{control}\right)}\times 100$$

### 2.9. In Vivo Studies

#### 2.9.1. Animals

The pharmacokinetic experiments were performed on male Wistar rats. The Institutional Animal Ethics Committee (IAEC), Anna University, Chennai, India approved the study protocol (Approval No. 1338). The animals were purchased from DORENCAMP, Bharathidasan University Animal House Capacity (IAEC/2021-2022; dated 28 August 2021). Utilizing Ehrlich’s ascites model on Balb/c mice, tumor regression activity was assessed. The animals were kept in an animal house that was registered with CPCSEA (2276/RO/24/p/CPCSEA). IAEC authorized the protocol, which has the protocol number IN/TN/IAEC/2018/07. Animals were kept in normal laboratory cages made of polypropylene, two to a cage with unrestricted access to a normal laboratory diet and water (ambient temperatures of 25 ± 2 °C and 55 ± 5% relative humidity).

#### 2.9.2. Pharmacokinetic Evaluation

Each of the two groups of animals contained six total individuals. One group received the ALT-HA-NPs (dispersed in PBS) for the studies, whereas the second group received the pure ALT (dispersed in PBS). Half a milliliter of the dispersions was administrated through the tail vein by intravenous administration. Each animal was given 0.2 mL diethyl ether anesthetic before the blood samples were taken after the retro-orbital plexus and immediately preserved within EDTA vacationers. The blood was withdrawn at the subsequent intervals: 0, 5, 15, 30, and 45 min, and 1, 2, 4, 6, 12, and 24 h. The blood-containing samples were then centrifuged at 4 °C for 10 min using a 5000-rpm machine (Eppendorf). Before further estimation, in Eppendorf tubes, the supernatant was collected and kept at −20 °C. The analysis method used by HPLC was modified from that used by Mohamed et al. [26]. The C_max_, T_max_, AUC_(0-t)_, t_1/2_, and the elimination rate constant was computed using the Microsoft Excel Addins-PK Solver and Pk1Pk2. To calculate relative bioavailability (Bio_rel_), Equation (6) was used:

(6) $${\mathrm{Bio}}_{\mathrm{rel}}=\frac{{\mathrm{AUC}}_{\left(\mathrm{test}\right)}\times {\;D}_{\left(\mathrm{test}\right)}}{{\mathrm{AUC}}_{\left(\mathrm{Std}\right)}\times {\;D}_{\left(\mathrm{Std}\right)}\;}$$

where, AUC—Area under the Curve; D—dose.

#### 2.9.3. Tumor Regression Evaluation

The Ehrlich’s ascites (EAC) model in Balb/c mice was used to test the in vivo anticancer activity. Subcutaneously, the EAC cells (100 µL) were injected into the Balb/shaved dorsal surface. Seven days after the inoculum, measurable tumors formed (0.08 cm^3^). The mice were separated into four groups (n = 6) after the initial tumor development. PBS was injected into the first group as a control, ALT-HA-NPs was injected into the second group once every two days (1.5 mg/200 µL), free ALT was injected into the third group once in two days/mice (129 µg/200 µL), and HA-NPs were injected into the fourth group once every two days (1.5 mg/200 µL). Each group received intravenous care through a tail vein. Every other day, vernier calipers were used to measure the tumor length and width, and Equation (7) was used to compute the tumor volume [27].

(7) $$\mathrm{Tumor}\;\mathrm{volume}\;\left(\mathrm{TV}\right)=0.5\times \;\mathrm{Length}\;\times {\;\mathrm{Width}}^{2}\;$$

The anti-tumor activity was then assessed in each case after measuring the tumor volume up to the 21st day.

### 2.10. Statistical Analysis

The mean and standard deviation of the data are the results of statistical analysis (mean ± SD). GraphPad (2018) Prism and Microsoft Excel (2019) were used to create bar graphs. ANOVA test was used to assess the significant differences among various experimental groups.

## 3. Results and Discussion

The most popular method for the preparation of HA-NPs was wet chemical precipitation from a solution, which has several benefits including a straightforward processing route, a high yield, being appropriate for industrial manufacturing, using cheap reagents, and the ability to create products with variable phase composition.

**Figure 1:**
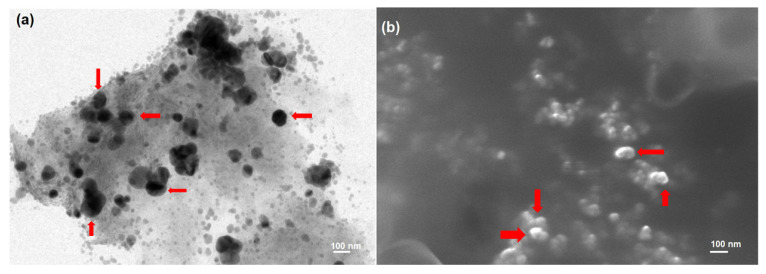
(**a**) TEM image of the ALT-HA-NPs and (**b**) SEM image of ALT-HA-NPs image, red arrows indicate NPs.

The prepared ALT-HA-NPs exhibited high EE% (96.27 ± 6.78%) and had drug loading of 8.2%. The particle size of the prepared ALT-HA-NPs was small in the nano range (156 ± 10.86 nm). The surface morphology of the prepared ALT-HA-NPs was examined by TEM and SEM. The prepared nanoparticle appeared spherical, as shown in Figure 1. Similar results were obtained by Ansari et al., who developed hydroxyapatite nanoparticles containing epirubicin for the treatment of cancer and examined the prepared nanoparticles with TEM [20]. Additionally, Soleimani et al. prepared Hydroxyapatite Nanoparticles for a human breast adenocarcinoma cell line and fibroblast examined the morphology with SEM and found that the prepared hydroxyapatite nanoparticles appeared spherical [28].

### 3.1. In Vitro Release Outcomes

**Figure 2:**
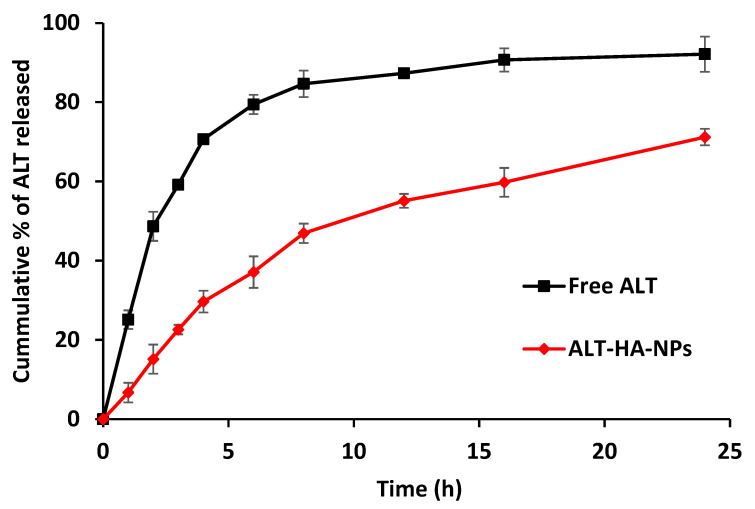
Cumulative percentage of ALT released from pure ALT and the ALT-HA-NPs (n = 3, mean ± SD).

The ALT in vitro release profile revealed a 24 h cumulative percentage of drug release up to 92.14 ± 4.45%. The ALT-HA-NPs, on the other hand, displayed a biphasic drug release profile, through an initial burst release up to 22.6 ± 1.2% in the initial 3 h, tailed by a continuous release (Figure 2). This first burst release of the ALT may be attributed to the drug molecules adhering to the nanoparticle surface [29]. The initial quick release may also be attributed to the nanoparticles’ great surface-to-volume ratio [30]. The drug is confined inside the orifices of the porous nanoparticles, which results in the second phase of slower sustained release. In 24 h, as much as 71.2 ± 2.1% of the drug was released. An extended length of time will be favored by the therapeutic impact of gratitude to such a slow and steady drug release, which has been documented by Kumar et al. (2015) [31]. Similar results were obtained by Cai et al. who developed zoledronic acid-functionalized hydroxyapatite-loaded polymeric nanoparticles for the treatment of osteoporosis and observed that the drug release displayed a biphasic drug release profile [29].

### 3.2. Hemolysis Outcomes

**Figure 3:**
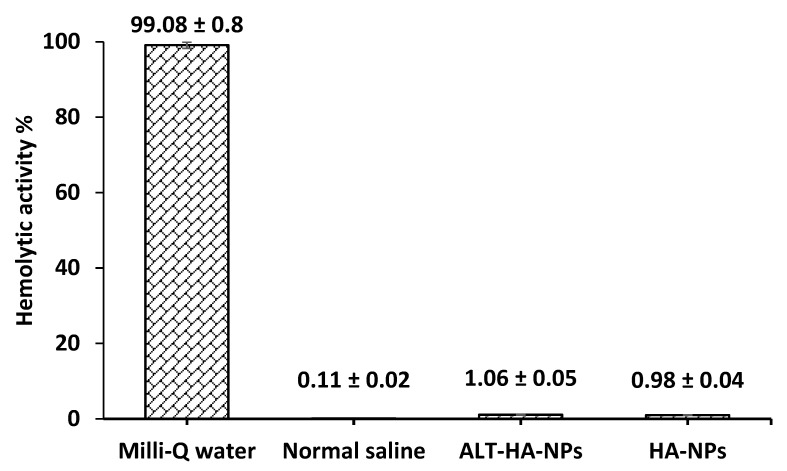
Hematolytic activity % for Milli-Q water, normal saline, ALT-HA-NPs, and HA-NPs (mean ± SD, n = 3, *p* <0.05).

To evaluate the NP biocompatibility and demonstrate their safety as a brand-new ALT carrier that satisfies preclinical requirements, a toxicity investigation was carried out. Wide-ranging hemolysis was seen in the case of Milli-Q water; however, isotonic saline was found to be minor. The ALT-HA-NPs had a hemolytic activity of 1.06 ± 0.05% and the unloaded HA-NPs had a hemolytic activity of 0.98 ± 0.04%, respectively (Figure 3). As a result, the NPs show no detectable hemolytic incompatibility (*p* < 0.05).

### 3.3. Cell Line Studies

#### MTT Assay

**Figure 4:**
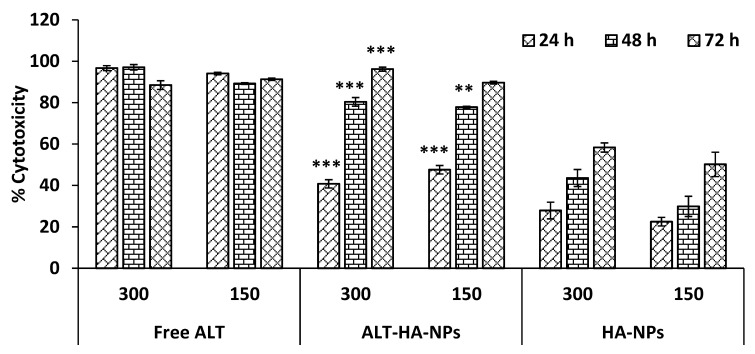
The outcome of ALT-HA-NPs, free ALT, and HA-NPs on the JURKAT cell line (n = 3, mean ± SD) is time- and dose-dependent. *** *p* < 0.001 and ** *p*< 0.01 denotes a significant difference between free ALT and ALT-HA-NPs.

At three different time intervals, 24, 48, and 72 h, the cytotoxicity was assessed in a dose- and time-dependent method using dual doses, i.e., 300 and 150 µg·mL^−1^. At a concentration of 300 (µg·mL^−1^), the cytotoxicity percent of the free ALT, ALT-HA-NPs and HA-NPs were reported to be 96.7 ± 0.22%, 40.8 ± 1.99%, and 27.9 ± 4.01%, whereas at a concentration of 150 µg·mL^−1^, the percentages were 94.1 ± 0.61%, 47.6 ± 2.03%, and 22.5 ± 2.08% after 24 h of treatment (Figure 4). At a high concentration of 300 µg·mL^−1^, free ALT, ALT-HA-NPs, and HA-NPs, respectively, demonstrated 97.1 ± 0.33%, 80.4 ± 2.08%, and 43.6 ± 4.11% cytotoxicity after 48 h of treatment, although at a low concentration of 150 µg·mL^−1^, 89.2 ± 0.41%, 77.8 ± 0.47%, and 29.9 ± 4.88% cytotoxicity was found. After 72 h of treatment, the percent cytotoxicity for the free ALT, ALT-HA-NPs, and HA-NPs, respectively, was observed to be 88.5 ± 2.11%, 96.2 ± 0.96%, and 58.36 ± 2.22%, respectively, whereas at 150 µg·mL^−1^, the stated cytotoxicity percent values were 91.3 ± 0.53%, 89.66 ± 0.67%, and 50.2 ± 5.89%. The drug loading in HA-NPs is 8.2%. As a result, the ALT-HA-NPs can produce maximum drug concentrations of 20.7 μg·mL^−1^ and 10.4 μg·mL^−1^, respectively, at concentrations of 300 and 150 μg·mL^−1^. We found that the ALT-HA-NPs can produce a similar outcome as the free ALT at a concentration of 1/10th when compared to the concentrations of the free ALT tested (300 and 150 μg·mL^−1^). The drug 24 h IC_50_ was determined to be 32.44 μg·mL^−1^ for free ALT, 15.54 μg·mL^−1^ for ALT-HA-NPs, and 497.11 μg·mL^−1^ blank HA-NPs. However, ALT-HA-NPs showed that the cytotoxicity was markedly enhanced (*p* < 0.01) as compared to the free ALT (control).

The results of the hemolytic study, the cytotoxicity experiment (MTT assay), and a further identically high IC_50_ value confirm the spherical-shaped HA-NPs exceptional biocompatibility and non-antigenicity, which were previously described in the literature [32,33]. When HA-NPs dissolve and release calcium ions into the cytoplasm after entering the cancer cell, it is believed that this disturbs intracellular calcium homeostasis. Calcium homeostasis is crucial because calcium plays a vital function as a secondary messenger, regulating crucial cellular processes including apoptosis and cell proliferation [34].

### 3.4. In Vivo Animal Model

#### 3.4.1. Pharmacokinetic Parameters

**Figure 5:**
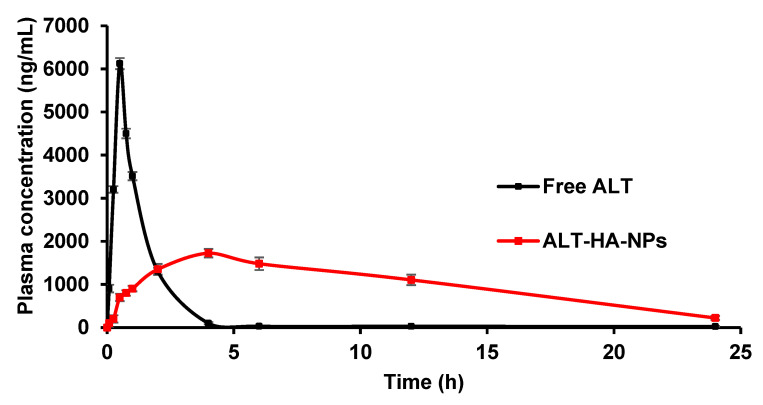
Plasma concentration–time curve after the intravenous injection of free ALT and ALT-HA-NPs in male Wistar rats (*p* < 0.001, mean ± SD, n = 3).

Pharmacokinetic investigations were carried out, and many parameters were evaluated using a non-compartmental study to support our findings. Figure 5 shows the drug levels detected in blood for the mixture of lyophilized drug concentrate and ALT-HA-NPs. Calculations were made based on the data to determine T_max_, C_max_, AUC_(0-t)_, drug half-life, elimination rate constant, and relative (Bio_rel_) bioavailability.

For the free ALT and the ALT-HA-NPs, the C_max_ was discovered to be 6124.4 ± 128.34 ng/mL and 1724.73 ± 98.46 ng/mL, respectively (*p* < 0.001). The T_max_ was determined to be 0.5 ± 0.24 h for the free ALT and 4 ± 0.31 h for the ALT-HA-NPs. The ALTs’ AUC in solution was determined to be 8243.11 ± 6457.51 ng. h/mL, but when it was loaded into HA-NPs, it was much higher at 19,998.71 ± 8653.421 ng·h/mL (*p* < 0.001), increasing the ALTs’ bioavailability by about 2.5 times. Drug-loaded HA-NPs were shown to have a significantly lower elimination rate constant (0.08756 ± 0.00279) than the free ALT (0.924567 ± 0.023456), and their half-life was found to be higher at 3.2 ± 0.29 h compared to the free ALTs of 0.87 ± 0.22 h.

ALT was thought to be rapidly eliminated from the body and to have a short half-life, which was well supported by Weber et al. (2015) [35]. As a result, it is clear from comparing the kinetic profiles of the two situations that the ALT was released from nanoparticles in a sustained manner and stays in the body for long time. The HA-NPs maintenance of constant plasma drug levels supports the increased T_1/2_ values. Comparing the AUC_(0-t)_ values with the extended T_max_ values made it clear that the body was exposed to the drug for a longer amount of time in the case of the drug-loaded HA-NPs. Enhancing bioavailability has advantages. Therefore, the HA nanoparticles of ALT are the best choice for safer therapy with increased drug bioavailability. To make it clinically feasible, further safety and efficacy studies are needed.

#### 3.4.2. Tumor Regression Studies

**Figure 6:**
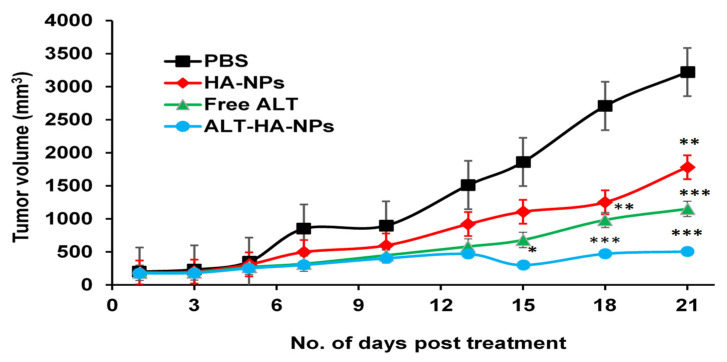
Anti-tumor activity of free ALT, ALT-HA-NPs, and blank HA-NPs (mean ± SD, n = 3). * significant compared to Control (PBS) group at *p* < 0.05, ** at *p* less than 0.01, *** at *p* less than 0.001.

Experiments on the 21-day tumor regression in Balb/c mice used as Ehrlich’s ascites model were effective. As expected, the tumor volume was shown to significantly grow in the control group; after 21 days, an average tumor volume of 3221.45 mm^3^ was documented. However, the tumor volume was significantly reduced in the other three groups (Figure 6). The ALT-HA-NPs produced the greatest reduction in tumor volume, with an average end volume of 506.25 mm^3^ (*p* < 0.001). However, the average final tumor volume in the free ALT was reported to be 1151.05 mm^3^ (*p* < 0.01). Furthermore, it was discovered that the blank HA-NPs significantly the reduced tumor size (1782.03 mm^3^). Overall, the tumor decreased to 1/6th of its volume after being treated with ALT-HA-NPs, compared to 1/2 of its volume when treated with the free ALT and blank HA-NPs (Figure 6). The cellular uptake study thoroughly demonstrated that the nanoparticles can be readily internalized by the cells, and these findings further support the possibility of a synergistic interaction between the anticancer ALT activity, and the calcium ions released from the carrier, which would result in greater tumor volume reduction than with the anticancer drug alone or with blank HA-NPs [36]. Additionally, it was hypothesized that the drug-loaded NPs enhanced the permeation and retention mechanism, which causes them to collect and localize in the tumor area, where they reduce the tumor volume.

## 4. Conclusions

According to our investigations, the ALT-HA-NPs exhibit superior combined anticancer effects compared to the free drug and blank HA-NPs. Due to its polar surface, HA with nanoscale dimensions is ideally adapted to interact with biomolecules in tissues and cells as well as with drugs. The cells may quickly ingest HA-NPs, and as they degrade, more Ca^2+^ eventually finds its way inside the cells. Altretamine anti-tumor action is then enhanced by calcium ion-induced proapoptotic pathways. It is necessary to conduct more research in this area to learn more about this synergism and its possible applications. Our investigation clearly shows that the formulation prepared can be used to treat CLL very successfully, minimizing the side effects such as diarrhea, weakness, nausea, and vomiting. For the research to advance, it is more important than ever to have a thorough understanding of the biomolecular effects of HA-NP-based systems on various cancer types. The experimental work undertaken for this purpose is quite complex, requiring the cooperation of biomolecular chemists, biomaterials scientists, oncologists, and biologists, in addition to the evaluation of full in vitro/in vivo toxicity profiles.

## Data Availability

Data are available on request.
